# Supplementary material for: Sr[Li2Al2O2N2]:Eu2+—A high performance red phosphor to brighten the future
Source: Nat Commun. 2019 Apr 23;10:1824. doi: 10.1038/s41467-019-09632-w (PMC6478936; doi:10.1038/s41467-019-09632-w)
Supplement: Supplementary file 1 — Supplementary Information [file 41467_2019_9632_MOESM1_ESM.pdf]

Supplementary Information

$Sr[Li_2Al_2O_2N_2]:Eu^{2+}$ – A high performance red phosphor to brighten the future

Gregor J. Hoerder[1], Markus Seibald[2], Dominik Baumann[2], Thorsten Schröder[2], Simon Peschke[2], Philipp C. Schmid[2], Tobias Tyborski[2], Philipp Pust[3], Ion Stoll[3], Michael Bergler[3], Christian Patzig[4], Stephan Reißaus[4], Michael Krause[4], Lutz Berthold[4], Thomas Höche[4], Dirk Johrendt[5], & Hubert Huppertz[1*]

Supplementary Figures

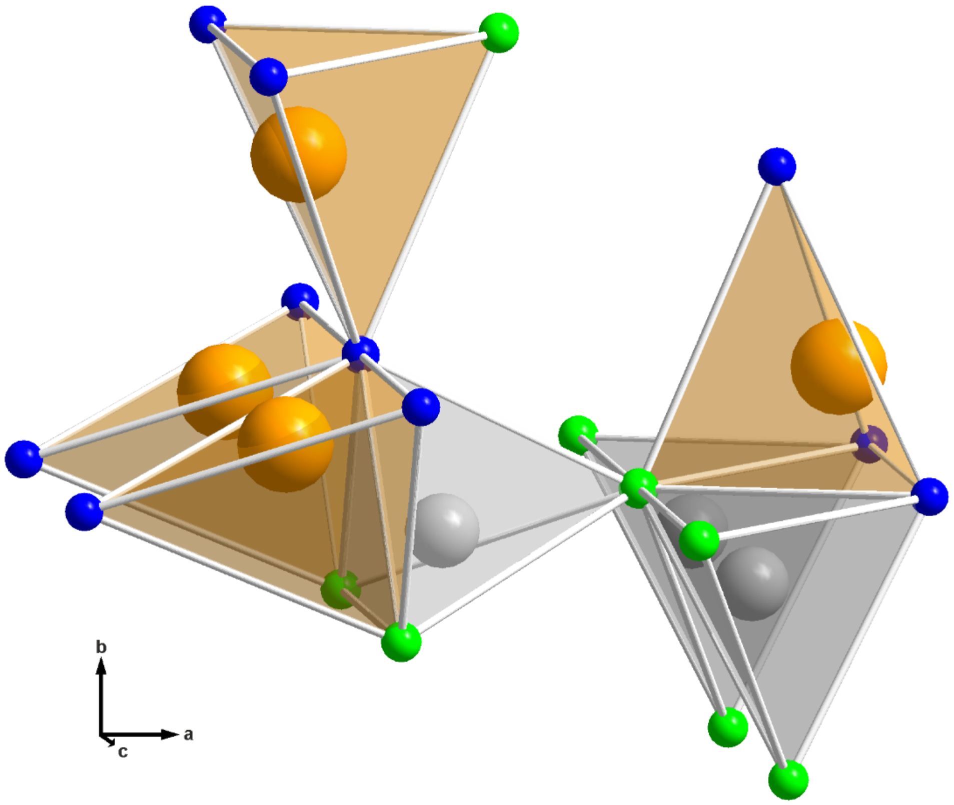

Supplementary Figure 1: Detail of the anionic network illustrating its connectivity. *Detail of the anionic network illustrating the fourfold bridging character of the nitrogen and oxygen atoms. Blue spheres represent oxygen, green spheres nitrogen, orange spheres lithium, and grey spheres aluminium atoms. The polyhedra are shown in the colour of their respective central atoms.*

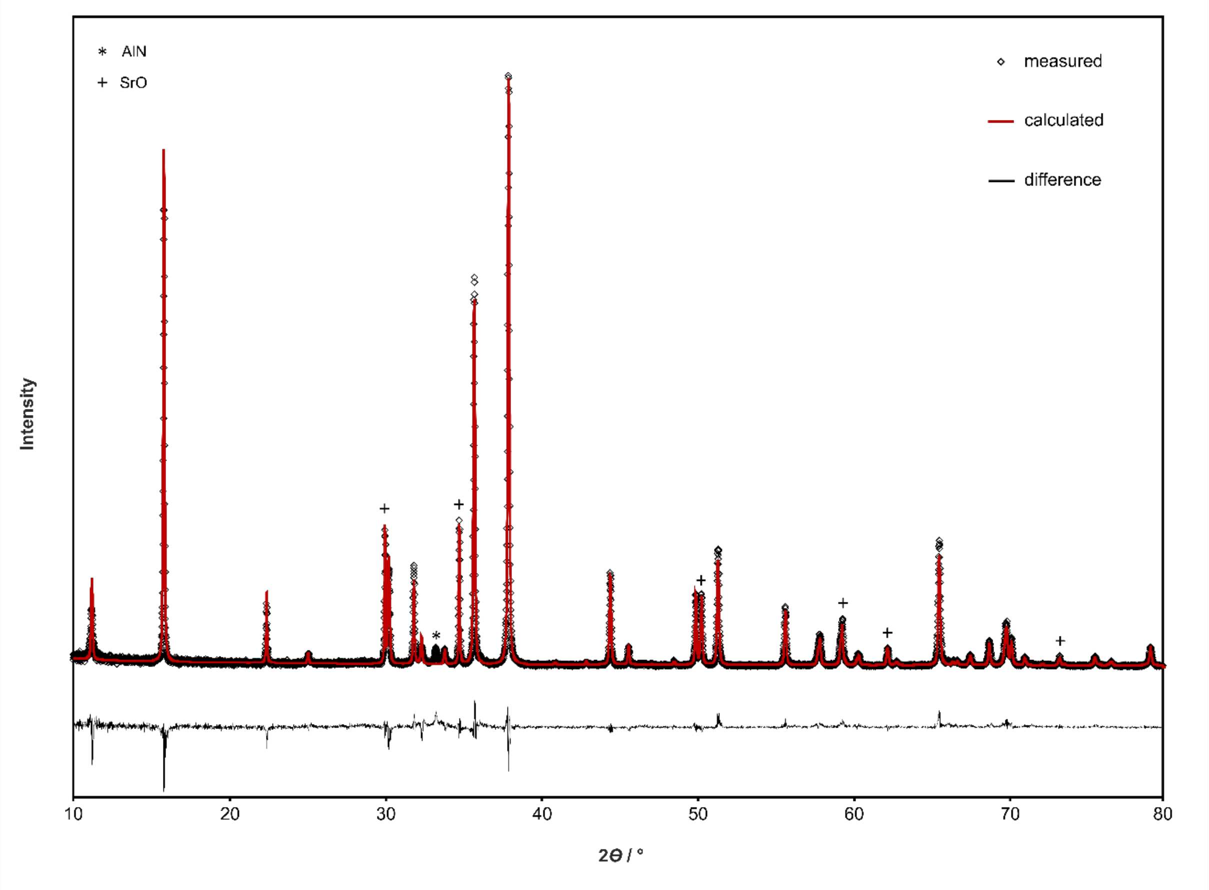

Supplementary Figure 2: Rietveld plot displaying a bulk sample with SrO (+) and AlN (*) impurities. *Rietveld plot displaying a bulk sample measurement of SALON (diamond shapes) in comparison with the data calculated via the Rietveld refinement (red line) with SrO (+) and AlN (*) impurities as well as the difference curve (black curve).*

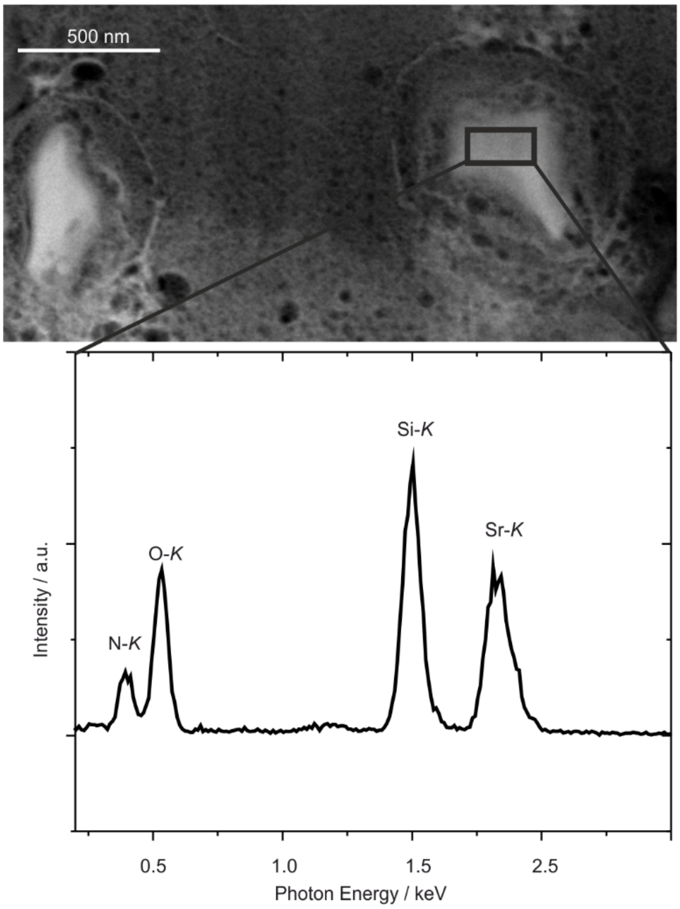

Supplementary Figure 3: Scanning transmission electron microscopy (STEM) and energy dispersive X-Ray spectroscopy (EDX). *Top: High-angle annular dark field STEM micrograph of two crystals within matrix material. Bottom: EDX spectrum of the crystal area as indicated in the STEM micrograph.*

.

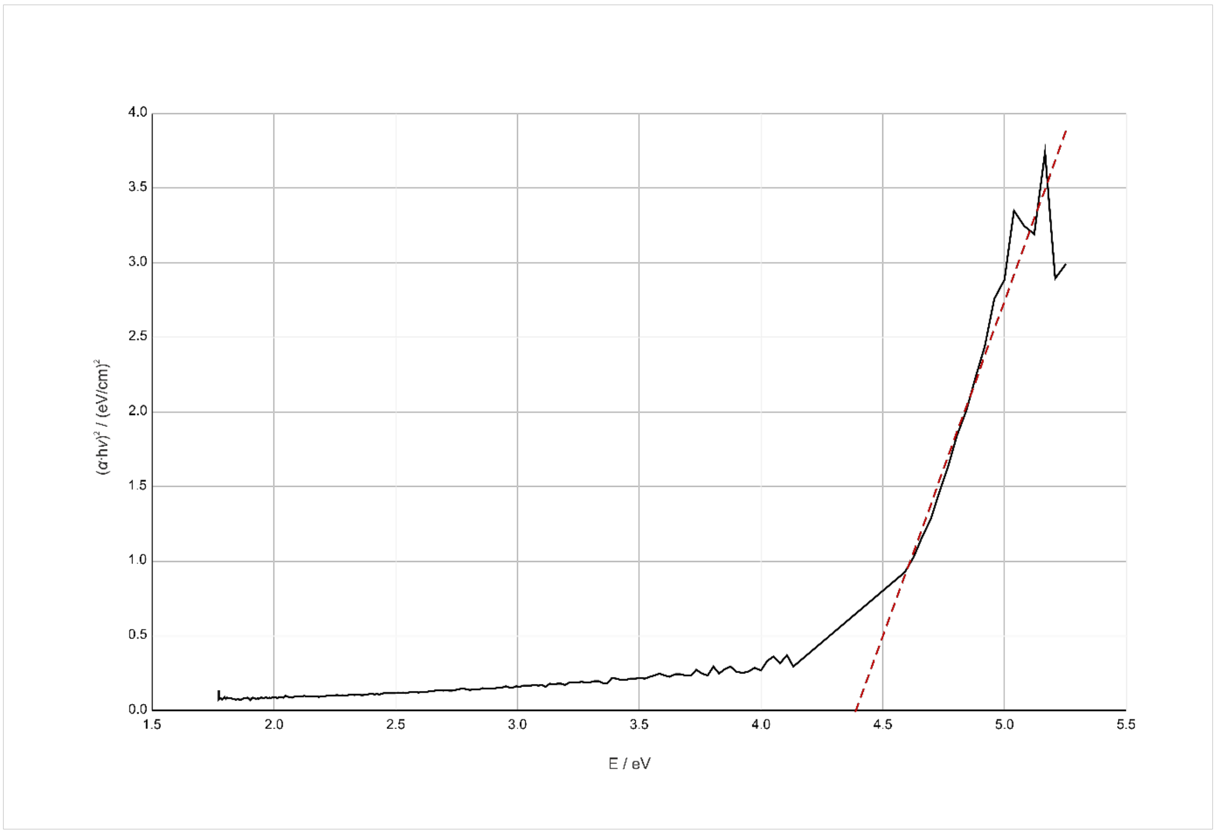

Supplementary Figure 4: Tauc-Plot for an undoped sample of $Sr[Li_2Al_2N_2O_2]$. *The measured data is displayed in black and the fitted function in red.*

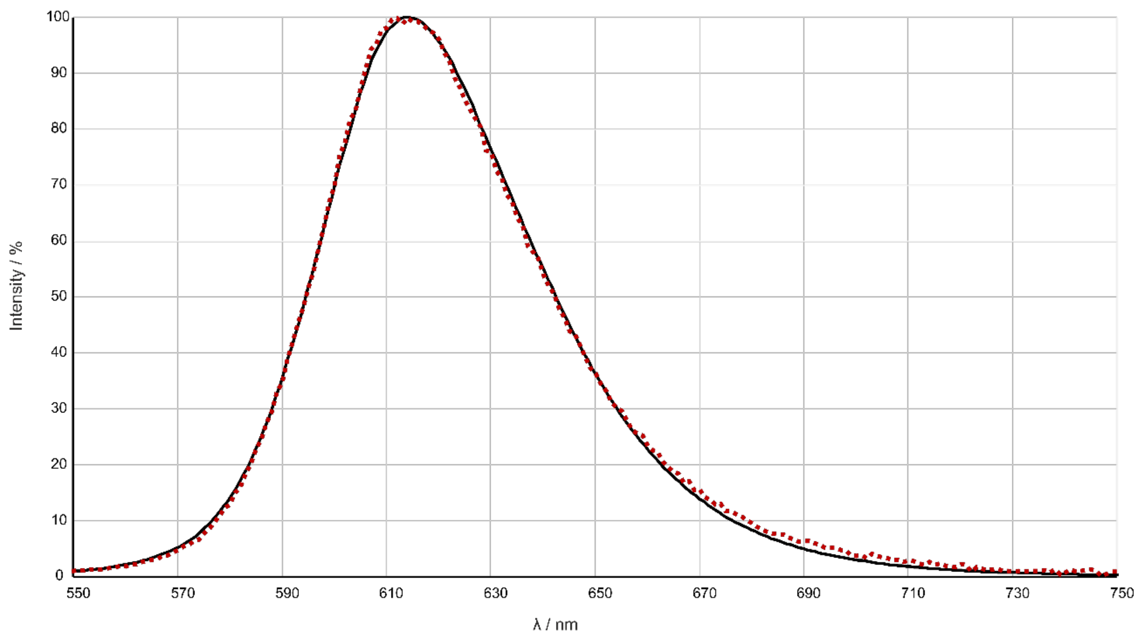

Supplementary Figure 5: Emission spectra of a bulk sample (dotted red) and a single-crystal (black line) of SALON.

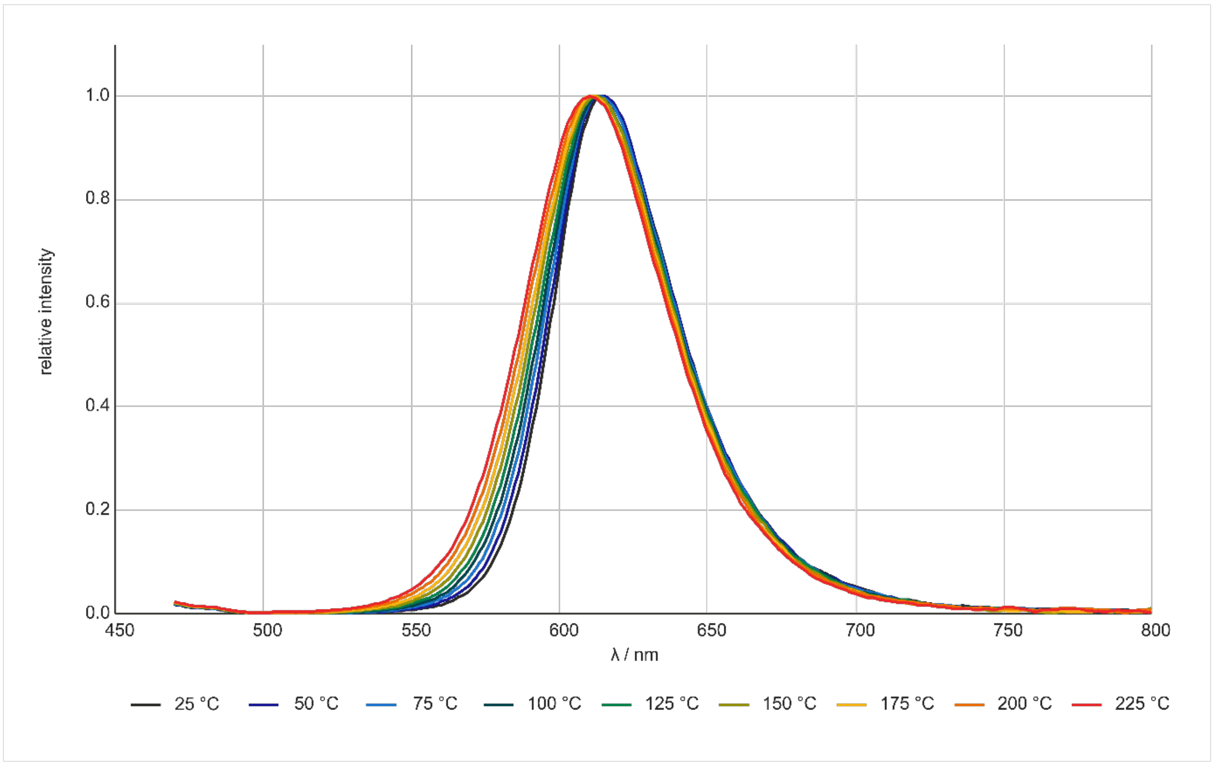

Supplementary Figure 6: Emission spectra of SALON between 25 °C and 225 °C in normalized intensities.

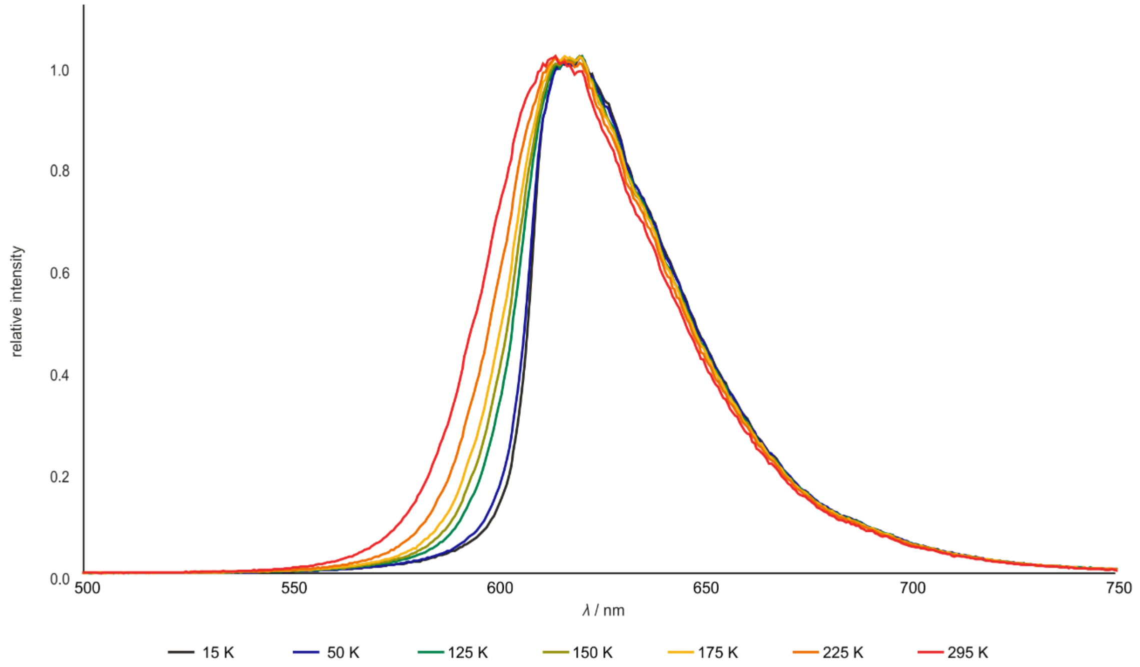

Supplementary Figure 7: Normalised photo luminescence measurements of SALON from 15 K to 295 K.

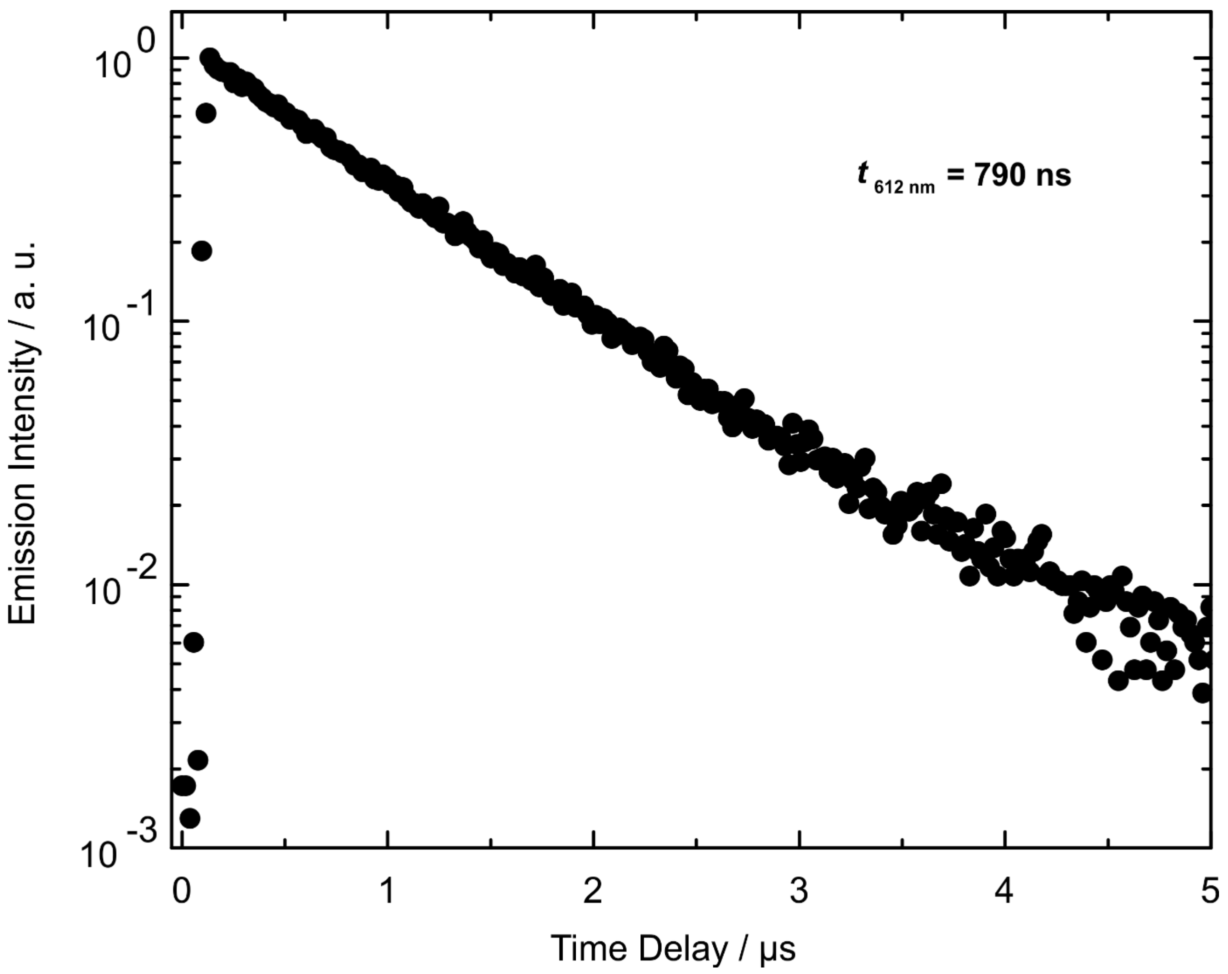

Supplementary Figure 8: Time-Correlated Single Photon Counting measurement of SALON.

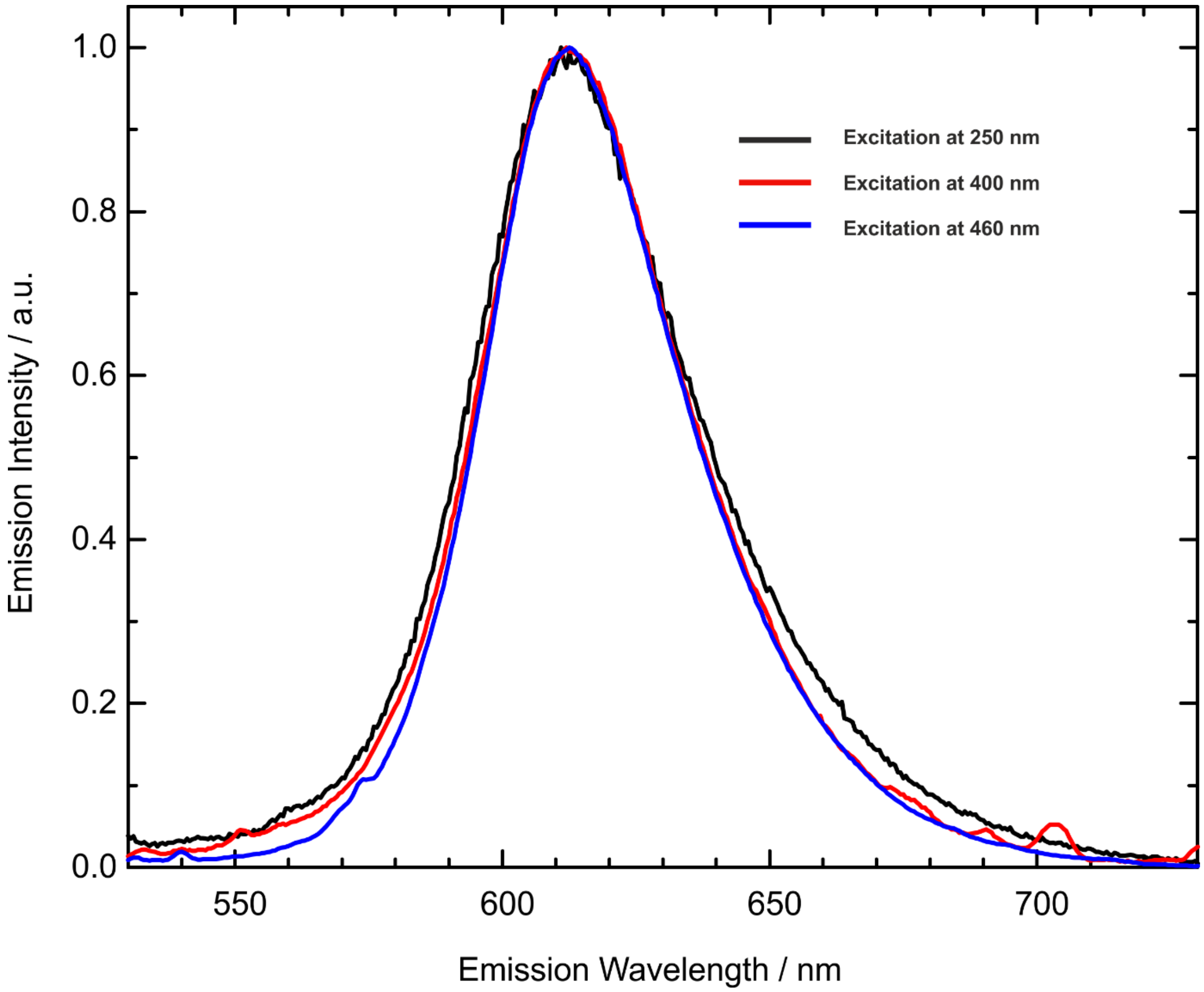

Supplementary Figure 9: Normalised emission spectra of SALON at different excitation wavelengths.

Supplementary Tables

Supplementary Table 1: Crystal structure refinement data.

Empirical formula	$Sr[Li_2Al_2O_2N_2]$
Formula weight / g mol^{-1}	215.48
Temperature / K	296(2)
Crystal system	tetragonal
Space group	$P4_2/m$
a / Å	7.959(2)
c / Å	3.184(2)
Volume / Å^3	201.7(2)
Z	1
ρ_{calc} / g cm^{-3}	3.548
μ / mm^{-1}	21.721
F(000)	200
Crystal size / mm^3	0.010 × 0.007 × 0.005
Radiation	Cu-K_α (λ = 1.54178 Å)
2Θ range for data collection / °	11.1 to 143.7
Index ranges	$-9 \le h \le 9$, $-9 \le k \le 9$, $-3 \le l \le 3$
Reflections collected	1534
Independent reflections	234 [R_{int} = 0.0490, R_σ = 0.0290]
Data/restraints/parameters	234/0/26
Goodness-of-fit on F^2	1.189
Final R indices ($I \ge 2\sigma(I)$)	R_1 = 0.0336, wR_2 = 0.0806
Final R indices (all data)	R_1 = 0.0424, wR_2 = 0.0857
Residual electron density/ e Å^{-3}	0.66/-0.75

Supplementary Table 2: Fractional atomic coordinates and isotropic displacement parameters /Å^2 for Sr[$Li_2Al_2O_2N_2$].

**Atom**	***Wyckoff position***	***x***	***y***	***z***	U_{eq}
Sr	2*c*	½	0	½	0.0180(4)
Al	4*j*	0.3533(3)	0.6842(3)	0	0.0167(5)
O	4*j*	0.2546(6)	0.8935(6)	0	0.017(2)
N	4*j*	0.5855(7)	0.7299(7)	0	0.014(2)
Li	4*j*	0.131(2)	0.821(2)	½	0.004(2)

Supplementary Table 3: Anisotropic displacement parameters /Å^2 for Sr[$Li_2Al_2O_2N_2$].

**Atom**	U_{11}	U_{22}	U_{33}	U_{23}	U_{13}	U_{12}
Sr	0.0160(5)	0.0133(5)	0.0247(6)	0	0	-0.0007(3)
Al	0.013(1)	0.012(1)	0.025(2)	0	0	-0.0014(8)
O	0.017(2)	0.009(2)	0.026(3)	0	0	0.000(2)
N	0.011(3)	0.013(3)	0.018(4)	0	0	0.002(2)

Supplementary Table 4: Interatomic distances /Å of Sr[$Li_2Al_2O_2N_2$] with standard deviations in parentheses.

Sr–O	2.659(4)	(4 ×)	Al–O	1.842(6)	Li–N	2.18(2)
Sr–N	2.760(5)	(4 ×)	Al–N	1.895(4)	Li–O	1.98(2)
			Al–N	1.895(4)	Li–O	1.960(7)
			Al–N	1.884(7)	Li–O	1.960(7)

Supplementary Table 5: Selected bond angles /° of Sr[$Li_2Al_2O_2N_2$] with standard deviations in parentheses:

O–Al–N	104.1(3)		N–Li–O	103.0(5)	
O–Al–N	103.1(1)	(2 ×)	N–Li–O	89.9(2)	(2 ×)
N–Al–N	115.0(1)	(2 ×)	O–Li–O	124.7(1)	(2 ×)
N–Al–N	114.3(1)		O–Li–O	108.7(1)	
O–Sr–O	73.6(1)	(2 ×)			
O–Sr–N	65.6(1)	(4 ×)			
O–Sr–N	73.8(1)	(4 ×)			
N–Sr–N	70.5(1)	(2 ×)			

Supplementary Table 6: Charges as calculated by means of bond-length/bond-strength and CHARDI

	Sr	Al	Li	N	O
∑V	+1.88	+2.88	+0.99	-3.02	-1.84
∑Q	+1.96	+3.11	+0.91	-2.74	-2.26

Supplementary Table 7: MAPLE values for all atoms in SALON

	MAPLE / $kJmol^{-1}$
Sr	1718
Al	5152
Li	796
N	5353
O	2130

Supplementary Table 8: *Comparison of the lattice parameters and cell volumes of the single-crystal and the Rietveld refinement.*

**Atom**	***Powder data***	***Single-crystal data***
Space group	*I*4/*m* (No. 87)	*I*4/*m* (No. 87)
a / Å	7.9508(2)	7.959(2)
c / Å	3.1907(1)	3.184(2)
V / $Å^3$	201.41(2)	201.7(2)

Supplementary Table 9: Charges as calculated using the Bader atom-in-molecules (AIM) approach.

Method	Sr	Li	Al	N	O
pDOS	+1.24	+0.83	+1.48	-1.65	-1.37
Bader	+1.49	+0.83	+2.35	-2.31	-1.62

Supplementary Notes

Supplementary Note 1: Rietveld Refinement

In addition to the single-crystal refinement, a Rietveld analysis was carried out on a bulk sample of Sr[$Li_2Al_2O_2N_2$]. As can be seen in Supplementary Table 8, the obtained lattice parameters are in good agreement with the single-crystal data and the overall goodness of fit for the Rietveld refinement is 1.5. The sample contained Sr[$Li_2Al_2O_2N_2$] with a purity of 93 wt%. The remaining 7 wt% of the sample could be identified as strontium oxide (which subsequently degrades upon contact with ambient air) and a non-quantifiable amount of aluminium nitride as indicated by a weak reflection at 2Θ equalling 33.2°. The impurities are most likely the result of a partial decomposition of SALON, which is discussed in the method section of the main manuscript. The data were collected using an Empyrean Powder Diffractometer (Panalytical, Netherlands) with Cu-$K_{\alpha1}$ radiation. The resulting Rietveld plot is displayed in Supplementary Figure 2.

Supplementary Note 2: Spectral Comparison

To ensure that the luminescence properties of the SALON single-crystal used for structure determination are similar to those of the bulk sample, a comparison of the respective emission spectra was conducted. As can be seen in Supplementary Figure 5, the two spectra match with only a small difference in the FWHM (0.9 nm), which can be attributed to reabsorption effects in the powdered sample.

Supplementary Methods

BLBS/CHARDI

Charge distributions were calculated according to the bond-length/bond-strength, BLBS [1] ($\sum V$), and the charge distribution, CHARDI[2] ($\sum Q$), concept. Within the limits of these concepts, the calculated charges are in good agreement with the expected values as shown in Supplementary Table 6.

MAPLE

In order to distinguish between oxygen and nitrogen, lattice energy calculations have been performed using the MAPLE[3,4] (Madelung part of lattice energy) approach. The partial MAPLE values of oxygen and nitrogen are expected to be within the range of 2100 to 2800 $kJmol^{-1}$ for oxygen and 5000 to 6000 $kJmol^{-1}$ for nitrogen, which allows for an unambiguous distinction. The values obtained for the oxygen and nitrogen positions in SALON yielded values, which fit perfectly within the expected range thereby verifying the proposed oxygen-nitrogen ordering. The results of the MAPLE calculations are listed in Supplementary Table 7.

Band gap measurement

The band gap was determined from diffuse reflectance spectra of an undoped sample using the Tauc-method[5], the corresponding plot can be seen below in Supplementary Figure 4. It should be noted that the Tauc-method can only provide an estimate of the band gap especially when the band gap is close to the detectable limit using a Xe-lamp for diffuse reflectance spectra. Additionally, the value is slightly affected by the side phase SrO. Yet, within the accuracy of the measurement the obtained value of 4.4 eV is in good agreement with the DFT calculation.

Thermal quenching behaviour

The thermal quenching was determined from emission spectra recorded between 25 °C and 225 °C from a powder sample of SALON. The measurements, expressed in normalized intensities, are displayed in Supplementary Figure 6. The thermal quenching resulted in a 4% drop of the integral intensity within the measured temperature range. At higher temperatures the spectrum broadens by 7 nm and the LER increases from 251 lm/W_{opt} to 302 lm/W_{opt} (ΔLER equal 51, +20%), which is caused by a blue shift of the emission maximum from 614 nm to 610 nm. For the reference material,

$Sr[LiAl_3N_4]:Eu^{2+}$, such an increase of the LER value is also reported (ΔLER equal 37, +48%, investigated in the range of 303 to 465 K).

LED-Measurement Setup for low temperature luminescence investigations

Cryogenic photoluminescence (PL) measurements were carried out in the temperature range from 15 to 295 K. The SALON phosphor powder was mixed with a solution of polymethyl methacrylate (PMMA, Sigma Aldrich) in dichloromethane to reach the desired concentration and dispersed onto a copper plate (10 x 10 x 0.3 mm) and hardened. The PMMA samples were mounted onto a copper holder with silver conductive paint to provide a good thermal connection. The holder was mounted onto a closed-cycle cold-finger helium cryostat (Leybold Vacuum Cryostat combined with a Coolpak 2000 Compressor). During all measurements, the samples were kept under vacuum (p less than 3 mbar) and the given temperatures correspond to the sample holder's bottom temperature. The samples were excited using a blue laser diode (OSRAM OS, λ_{max} equal 448 nm at 1.6 W power). As no transmission measurements are possible using a copper carrier, the samples were excited in reflection mode. The fluorescence was collected using free space optics, coupled into a fibre and recorded using a spectrometer (CAS 140 CT). To eliminate the excitation light from the detected signal, an optical notch filter (optical cut-off between 425 and 455 nm, CHROMA) was placed just in front of the fibre. The normalised emissions from 15 K to 295 K are displayed in Supplementary Figure 7. Due to a shift of the sample position relative to the excitation beam caused by the cooling process, the absolute emission intensities could not reliably be determined.

Excitation dependence of the emission spectrum

Emission measurements have been performed with the Spectrometer FSP920 (Edinburgh Instruments, UK) using a 450 W Xenon lamp. The spectral resolution is better than 2 nm and the red sensitive PMT (Photo-Multiplier Tube) R928P (Hamamatsu, Japan) has been used for photon detection. No significant additional emission bands have been measured under 400 nm and 250 nm excitation. Furthermore, a slight increase of the FWHM by about 5 nm can be observed for an excitation wavelength of 250 nm but no line emission is measured, which would be expected for Eu^{3+} emission.

Lifetime measurements

Time-resolved measurements have been performed with the spectrometer FSP920 (Edinburgh Instruments, UK) using a nf 900 nanosecond flash lamp. The spectral resolution was better than 2 nm

and the red sensitive PMT (Photo-Multiplier Tube) R928P (Hamamatsu, Japan) has been used for photon detection. Transients have been measured with TCSPC (Time-Correlated Single Photon Counting) with 512 channels, a maximal time delay of 10 µs and a repetition rate of 15 kHz. The nanosecond flash lamp was filled with pure hydrogen and emitted light impulses with a temporal width of less than 6 ns. A single exponential fitting procedure yielded a relaxation constant τ of 790 ns for SALON under 460 nm excitation, measured at 612 nm emission wavelength. Both, the linear shape of the transient in the log-plot and additional long-term measurements up to 50 µs (data not plotted) showed a complete relaxation of the excited states with only one time constant. Measured relaxation constants at emission wavelengths of 600 nm and 640 nm were slightly (a couple of 10 ns) smaller and larger, respectively (data not shown).

Supplementary References

1 Hoppe, R. *et al.* A new route to charge distributions in ionic solids. *J. Less-Common Met.* **156**, 105-122 (1989).

2 Nespolo, M. & Guillot, B. CHARDI2015: charge distribution analysis of non-molecular structures. *J. Appl. Crystallogr.* **49**, 317-321 (2016).

3 Hoppe, R. Über Madelungfaktoren. *Angew. Chem.* **78**, 52-63 (1966).

4 Hoppe, R. The Coordination Number – an "Inorganic Chameleon". *Angew. Chem. Int. Ed.* **9**, 25-34 (1970).

5 J. Tauc, R. G., A. Vancu. Optical Properties and Electronic Structure of Amorphous Germanium. *Phys. Status Solidi* **15**, 627-637 (1966).
